# Fovea-sparing internal limiting membrane peeling with inverted flap technique versus standard internal limiting membrane peeling for symptomatic myopic foveoschisis

**DOI:** 10.1038/s41598-024-53097-x

**Published:** 2024-01-30

**Authors:** Antonio Polito, Giulio Garruto, Emilia Maggio, Maurizio Mete, Massimo Guerriero, Grazia Pertile

**Affiliations:** 1 IRCCS Sacro Cuore Don Calabria Hospital, Via Don Sempreboni 5-Negrar, 37024 Verona, Italy; 2 Department of Cultures and Civilizations, University of Verona, 37134 Verona, Italy

**Keywords:** Diseases, Medical research

## Abstract

To study the long-term outcomes of standard ILM peeling and fovea sparing with inverted flap (FSIF) peeling for symptomatic myopic foveoschisis (MF). This retrospective observational study included 36 eyes of 34 consecutive patients who underwent vitrectomy with standard ILM peeling and FSIF peeling for MF between April 2012 and march 2020. The primary outcome measures included best-corrected visual acuity (BCVA) and central foveal thickness (CFT) at 1 month and final visit and postoperative development of macular hole. There were 14 eyes in the standard ILM peeling group and 22 eyes and in the FSIF peeling groups with a mean FU of 34.2 months (SD 23.3; min. 12–max. 96) and 27.7 months (SD 14.9; min. 12–max. 63), respectively. In both groups BCVA was not significantly improved at 1 month but improved at last visit from 0.55 ± 0.21 to 0.37 ± 0.29 in the standard ILM peeling group (P = 0.0154) and from 0.57 ± 0.27 to 0.28 ± 0.23 in the FSIF peeling group (P < 0.0001). At 1 month and final visit CMT decreased from 572 ± 183.5 µm to 277.5 ± 95.2 µm and to 250.4 ± 96.1 µm, respectively, in the standard ILM peeling group and from 589.9 ± 189.8 µm to 383 ± 110.1 µm and 162.3 ± 74.8 µm in the FSIF peeling group (P < 0.001 for both groups at both time-points). The preoperative and postoperative BCVA and CMT showed no significant differences between groups. Three of the eyes in the standard ILM peeling group developed postoperative macular hole at 1, 10, 24 months, respectively, and none of the eyes in the FSIF peeling group. Multivariate analysis revealed that a better BCVA was the only independent factor correlated with the final BCVA. In this study, standard ILM peeling and FSIF peeling were both beneficial in improving the anatomy and function of eyes with MF. Postoperative MH may occur up to 2 years after standard peeling and seem effectively prevented by FSIF peeling.

## Introduction

Myopic foveoschisis (MF) is a relatively stable condition and only few cases become symptomatic over time thus requiring surgery^1^. Factors associated with progression include axial length, chorioretinal atrophy at the posterior pole, steep posterior staphyloma, vitreomacular traction secondary to posterior vitreoschisis, rigidity of ILM and epiretinal tissue proliferation^2^. Patients are often unaware of this condition due to its silent and slow nature^3^. Optical coherence tomography (OCT) is the most reliable tool both to confirm the presence and to assess its extent and progression over time. When tractional forces increase, four major complications may occur, and patients become symptomatic. First, a foveal outer lamellar hole or a localized focal retinal detachment may develop^4,5^. If the area of detached retina further enlarges retinoschisis may resolve but vision declines^6^. Second, a full thickness macular hole may occur with or without a macular detachment^7^. Third an inner lamellar hole may develop, and this may either remain stable for long or progress into an outer lamellar hole or a full thickness macular hole^8^. Forth, tangential tractions from epiretinal tissue proliferation may increase thus inducing retinal folds and metamorphopsia even in the presence of relatively good visual acuity, as in macular pucker. OCT changes relating decreased vision or symptoms to foveoschisis progression, may guide us towards a surgical decision. In such cases ILM peeling may successfully relieve traction mechanism and improve both the anatomy and function^9^. However, development of a full thickness macular hole is a well-known complication after vitrectomy and refinements in the surgical technique have been recently reported for its prevention^10^. Since macular hole formation may derive from surgical trauma of a thinned central fovea by peeling of the ILM, new techniques avoiding complete ILM removal have been developed^11–13^. More recently, fovea sparing ILM peeling has been combined with the inverted flap technique to further reduce the risk of MH formation^14–16^. However, the long-term benefit of the combined technique remains unclear^17^.

The purpose of this study was to report the long-term effectiveness and safety of the combined fovea-sparing ILM peeling with inverted flap technique (FSIF peeling) and compare the results with those for standard ILM peeling in patients with symptomatic myopic foveoschisis.

## Results

Thirty-six eyes of thirty-four patients (14 eyes in the standard ILM peeling group and 22 eyes in the FSIF peeling group) were included. Table 1 summarizes patient demographics and preoperative ocular findings for the two groups. There were no significant differences between groups in age, sex, spherical equivalent, axial length, lens status, preoperative BCVA, MF type, CMT. The total follow-up duration was 34.5 ± 23.6 (9–96) months in the standard ILM peeling group and 26.1 ± 16.7 (6–63) months in the FSIF group.

**Table 1:** Patient characteristics.

	Standard ILM peeling (n eyes = 14)	Fovea-sparing with inverted flap ILM peeling (n eyes = 22)	P value
No. of eyes/no. of patients	14/13	22/21	n.a.
Age (yrs) mean (SD) [range]	59.4 (10.1) [42–76]	60.7 (12.2) [40–83]	0.359
Gender (male/female)	2/12	8/14	0.255
Preoperative LogMAR BCVA mean (SD)	0.55 (0.21)	0.57 (0.27)	0.392
Spherical equivalent mean (SD)	− 14.7 (6.2)	− 12.5 (6.6)	0.154
Axial length (mm) mean (SD)	30.7 (1.7)	30.6 (1.9)	0.444
Type of MF (MF-only/ILH/FD)	5/6/3	5/10/7	0.755
CFT (μm) mean (SD)	572.0 (183.5)	589.9 (189.8)	0.390
Lens status, no.			
Phakia	11	16	0.999
Pseudophakia	3	5	
Aphakia	0	1	
Surgery combined with phaco no. (%)	8 (57.1)	11 (50.0)	0.338
Follow-up (mo) mean (SD) [range]	34.7 (23.3) [12–96]	27.7 (14.9) [12–63]	0.148

**Figure 1:**
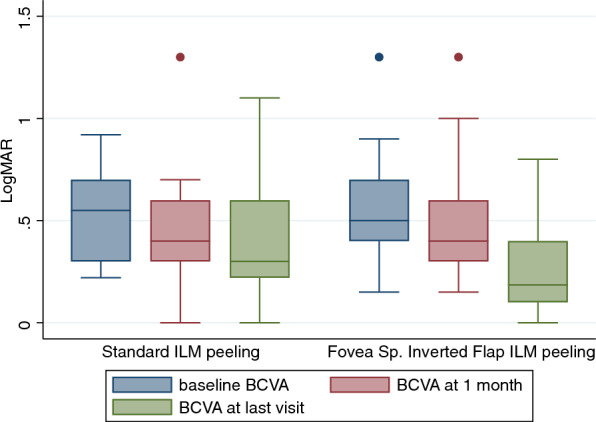
Functional results. The graph shows the best corrected visual acuity at baseline and follow up in the group treated with standard ILM peeling and in the group treated with fovea sparing with inverted flap ILM peeling.

We collected both the 1-month follow up data and last visit follow-up data (Table 2). LogMAR BCVA was not significantly improved at 1 month, but improved at last visit in both groups, from 0.55 ± 0.21 to 0.37 ± 0.29 in the standard ILM peeling group (P = 0.0154) and from 0.57 ± 0.27 to 0.28 ± 0.23 in the FSIF peeling group (P < 0.0001) (Fig. 1). Although there were no statistically significant differences between groups, the FSIF peeling group presented a greater gain in vision at last follow-up visit.

**Table 2:** Visual and anatomic outcomes after surgery.

	Standard ILM peeling (n = 14)	Fovea-sparing with inverted flap ILM peeling (n = 22)	P value
LogMAR BCVA at 1 month mean (SD)	0.47 (0.31)	0.50 (0.28)	0.382
LogMAR BCVA at final visit mean (SD)	0.37 (0.29)	0.27 (0.23)	0.157
Visual improvement at 1 mo mean (SD)	0.08 (0.24)	0.07 (0.25)	0.459
Visual improvement at final visit mean (SD)	0.18 (0.27)	0.29 (0.22)	0.095
Visual change at final visit no. (%)			
Improved ≥ 2 lines	6 (42.9)	14 (63.6)	0.301
No change	7 (50.0)	8 (36.4)	
Worsened ≥ 2 lines	1 (7.1)	0 (0.0)	
CFT at 1 mo. (μm) mean (SD)	277.5 (95.2)	383.0 (110.1)	0.002
CFT at final visit (μm) mean (SD)	250.4 (96.1)	262.3 (74.8)	0.349
Eyes with resolved MF at 1 mo, no. (%)	7 (50.0)	5 (22.7)	0.091
Eyes with resolved MF at last visit, no. (%)	12 (85.7)	18 (81.8)	0.999
Eyes with intact EZ at final visit, no. (%)	11 (78.6)	17 (77.3)	0.999
Postoperative MH formation, no. (%)	3 (21.4)	0 (0.0)	0.051

**Figure 2:**
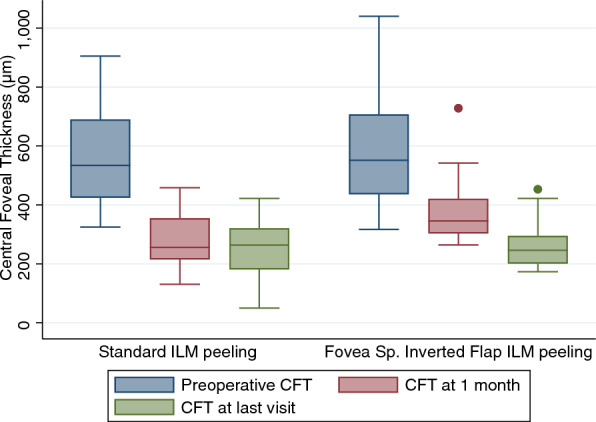
Anatomical results. The graph shows the central foveal thickness at baseline and follow up in the group treated with standard ILM peeling and in the group treated with fovea sparing with inverted flap ILM peeling.

**Figure 3:**
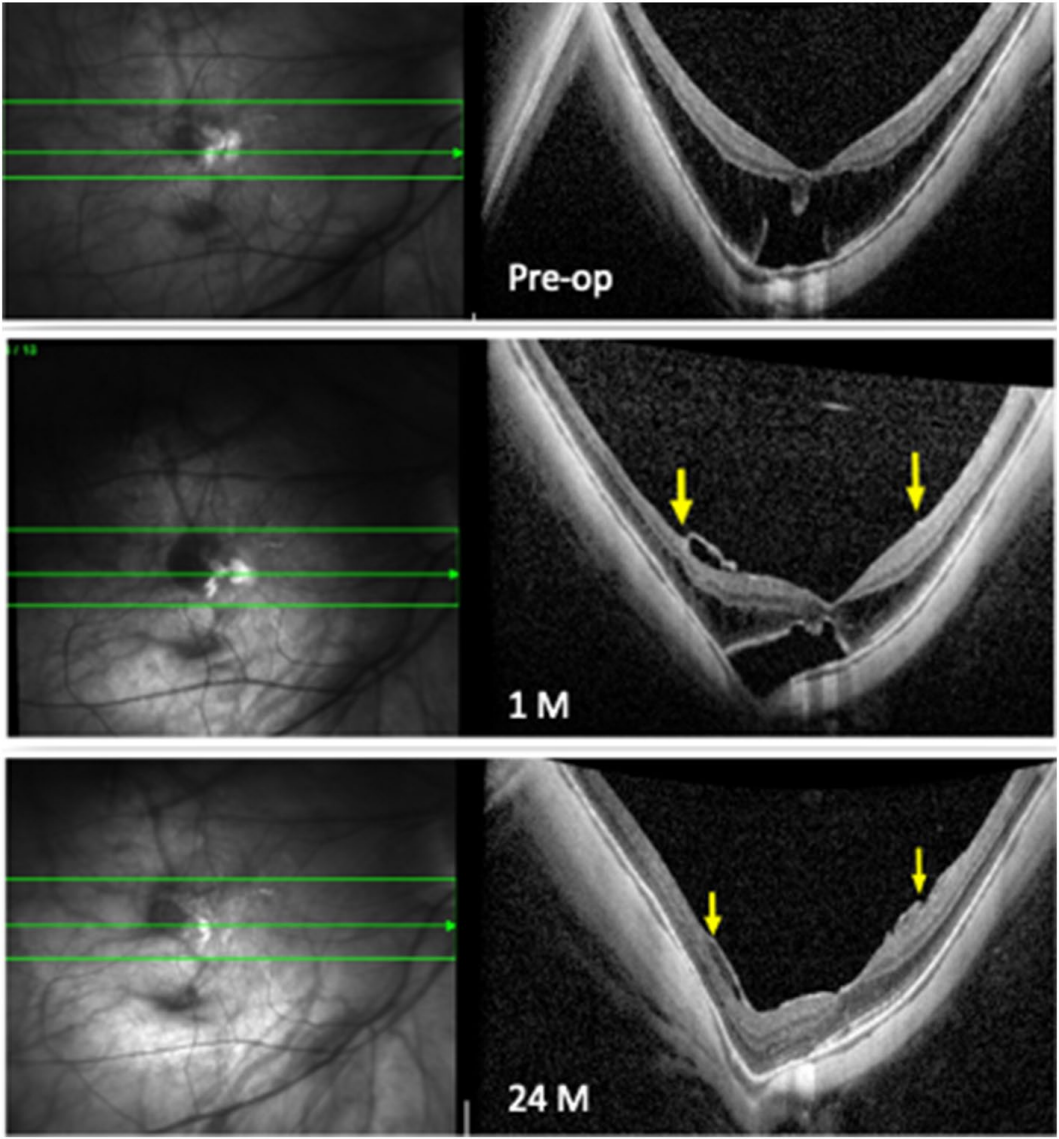
Raster lines comparison report of OCT images before and after surgery. Superior: preoperative image shows myopic foveoschisis with foveal retinal detachment; at 1 month after fovea sparing with inverted flap ILM peeling the foveoschisis is decreased and the foveal retinal detachment is reduced; the edges of the inverted ILM flap can be seen (yellow lines); at 2 years both foveoschisis and detachment are resolved, and the margin of residual ILM are still visible (yellow lines).

The BCVA improved by 2 or more lines for six eyes (43%) in the standard group and 14 eyes (64%) in the FSIF group (P = 0.301). However, a loss of 2 or more lines was detected for 1 eye in the standard ILM peeling group and none of the eyes in the FSIF peeling group. Myopic foveoschisis and foveal detachment (when present) completely regressed in 7 and 12 eyes of the 14 eyes of the first group at 1 month and final visit, respectively and in 5 and 18 eyes of the 22 eyes of the second group. An intact EZ was detected by SD-OCT in 11 (78%) eyes of the standard ILM peeling group and in 17 (77%) eyes of the FSIF peeling group. CMT decreased from 572 ± 183.5 µm to 277.5 ± 95.2 µm and to 250.4 ± 96.1 µm at 1 month and final visit, respectively, in the standard ILM peeling group and from 589.9 ± 189.8 µm to 383 ± 110.1 µm and 162.3 ± 74.8 µm in the FSIF peeling group (P < 0.001 for both groups at both time-points) (Fig. 2). Representative OCT images of the postoperative course of eyes that received FSIF peeling are shown in Fig. 3.

**Figure 4:**
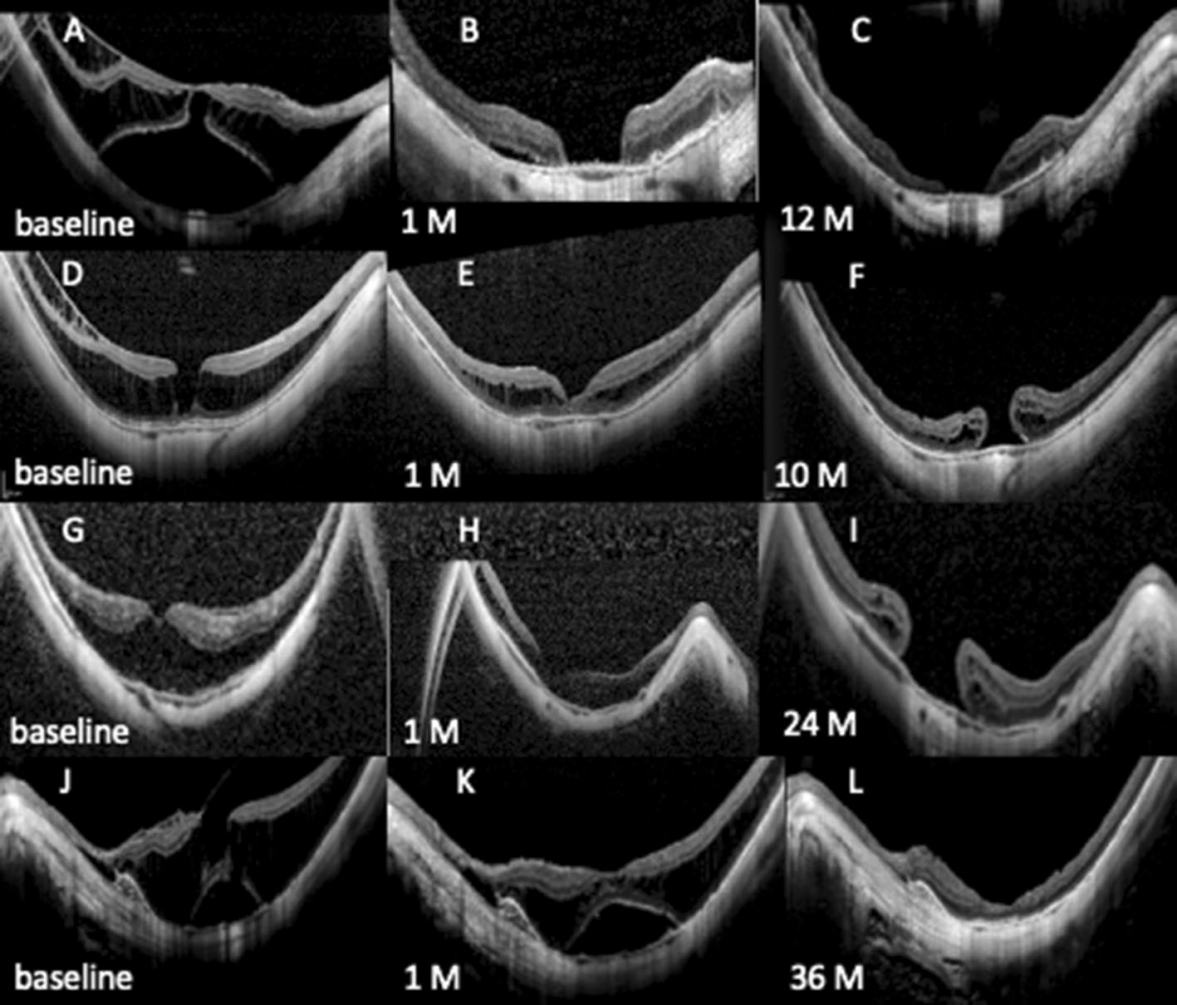
Three cases that developed a full-thickness macular hole (MH) in the standard ILM peeling group and the fellow eye of the first case who underwent uneventful FSIF peeling 4 years later. (**A**) Preoperative OCT image of the first case showing a foveal RD with outer lamellar MH and macular retinoschisis. (**B**) At 1 month after surgery, the retinoschisis and foveal RD are resolved, but a large a full-thickness MH is present. (**C**) At 1 year the full thickness MH is well visible with thinned margins. (**D**) Preoperative OCT image of the second case showing an inner lamellar hole and macular retinoschisis. (**E**) At 1 month after surgery retinoschisis is significantly reduced and foveal contour restored. (**F**) At 10 months after surgery a full thickness MH has developed. (**G**) Preoperative OCT image of the third case showing a myopic foveoschisis-only pattern. (**H**) At 1 month after surgery the retinoschisis is significantly improved with a normalized foveal contour. (**I**) At 2 years after surgery a large macular hole with thick margin is present. (**J**) Preoperative OCT image of the fellow eye of the first patient, presenting 4 years later with a foveal detachment with an inner lamellar hole and foveoschisis. (**K**) At 1 month after surgery the inner lamellar defect is no longer visible, but a foveal detachment persisted. (**L**) At 3 years after surgery both foveoschisis and detachment are completely resolved with achievement of anatomic resolution.

While 3 eyes developed a MH after standard peeling, no cases of postoperative MH were observed in the FSIF group. Among the eyes developing a MH, OCT showed 1 FD with outer lamellar defect, 1 ILH and 1 MF-only pattern at baseline (Table 3). The patient with preoperative FD developing a MH with atrophic changes and severe visual loss in the first eye underwent uneventful FSIF peeling in the fellow eye for FD with ILH four year later (Fig. 4). In this eye his vision improved from 0.4 to 0.1 LogMAR with a gain of 4 lines at 3.5 years.

**Table 3:** Characteristics of three patients with postoperative macular hole.

Patient	Age (yrs)	Gender	Type of MF	Axial lenght	Refractive error	Preoperative BCVA	Preoperative CFT	Time between surgery and MH	Second surgery	Final anatomic status	Postoperative BCVA at final visit
1	76	M	FD	28.6	− 12	0.8	905	1 M	None	Open	1.1
2	56	F	ILMH	29.8	− 17.25	0.2	509	10 M	Autologous full thickness retinal transplant	Open	0.3
3	56	F	MF-only	31.5	− 21.75	0.7	436	24 M	None	Open	0.6

Multivariate linear regression model revealed that a better preoperative BCVA value was the only factor significantly associated with a better BCVA at last visit (coef = 0.68, 95% CI = 0.30–1.07, P = 0.002). On the contrary age, sex, axial length, lens status, type of MF, type of surgery, preoperative CFT, postoperative EZ integrity and follow-up duration showed no correlation with postoperative BCVA.

## Discussion

Our study has demonstrated the effectiveness of both standard and combined FSIF ILM peeling techniques in achieving sustained vision improvement following surgery. With the FSIF ILM peeling technique, nearly two-thirds of patients experienced an improvement of 2 or more lines of vision due to the complete resolution of foveoschisis and the restoration of macular anatomy. Importantly, the FSIF ILM peeling technique did not result in macular hole (MH) formation, thereby enhancing the likelihood of visual improvement. In contrast, the standard ILM peeling approach failed to prevent MH formation in three cases (21%), leading to further visual deterioration.

Numerous previous studies have highlighted the potential of vitrectomy with complete ILM peeling for addressing symptomatic myopic foveoschisis and improving vision by relieving traction^9^. However, these studies have also reported a high rate of MH formation (ranging from 5 to 21%), particularly when a preoperative photoreceptor layer defect was present^12^. Consequently, in 2012, Shimada et al. proposed a novel technique known as "fovea-sparing" ILM peeling, which involves preserving the epi-foveal ILM to prevent mechanical traction and potential foveal breaks^11^. This approach resulted in a mean visual improvement of 0.3 logMAR in 15 eyes, which is comparable to our finding of 0.28 logMAR, and importantly, no postoperative MH cases were observed in our study. Since then, various studies have introduced slightly different surgical approaches for preserving the central ILM. Among these, Shiraki et al. in 2020 reported a long-term visual improvement of 0.28 logMAR in 25 eyes with no postoperative MH over an average follow-up of 21 months^17^. A recent meta-analysis, encompassing 10 studies comparing standard ILM peeling with fovea-sparing techniques, also demonstrated better visual acuity and lower rates of postoperative MH with the fovea-sparing technique^12^. While we did not observe a statistically significant difference in postoperative BCVA, possibly due to our sample size, we did notice a greater VA improvement (3 vs. 2 lines) in the FSIF group during the final visit.

Despite the growing consensus favoring fovea-sparing ILM peeling, recent reports have hinted at the potential for further refinement. Some authors have suggested that, particularly in cases with very thin fovea, such as outer lamellar holes with foveal detachment, the limited mechanical force applied during fovea sparing could still damage the extremely fragile tissue^15,18^. As a response to this concern, the combination with the inverted flap technique, well-established for treating large or persistent idiopathic MH or myopic MH, has been proposed^14,15,19,20^. This combined technique offers three significant advantages. First, it minimizes mechanical stress on the ILM during peeling, reducing the risk of trauma to the Muller cell cone and preserving foveal glial structure. Second, it leaves an ILM flap atop the fovea, in addition to the epifoveal tissue, further reducing tractional forces on the fovea and minimizing the possibility of inadvertent complete foveal ILM peeling. Third, the inverted flap may act as a glial scaffold for closing pre-existing, unrecognized, perioperative, or postoperative macular holes. Therefore, we adopted the combined technique from the outset when introducing fovea-sparing ILM peeling at our institution, which is why our historical control group underwent standard ILM peeling.

Lin and Yang recently demonstrated the success of combining fovea sparing with ILM flap in preventing macular MH in 35 eyes over a 20-month follow-up period^14^. In contrast, within their series, using the fovea sparing "only" technique, three out of 31 eyes developed post-operative MH, two immediately and one after 17 months. Zheng et al., who compared fovea sparing ILM surgery with and without the inverted flap, reported two cases of postoperative MH, one for each type of surgery. However, the MH in the non-inverted flap group required further surgery, whereas the inverted flap MH spontaneously closed at four months, presumably due to the presence of the flap^15^. More recently, Feng et al. compared standard and fovea-sparing ILM peeling with combined FSIF ILM peeling in eyes with MF and lamellar hole and found a lower risk of post-operative MH. They concluded that the ILM flap cover at the fovea may play a protective role for vitrectomy in eyes with an extremely thin fovea^16^.

In our study, MH opening was only observed in the standard ILM peeling group. Interestingly, only one patient developed a postoperative macular hole immediately after peeling, suggesting direct surgical trauma resulting from mechanical stretching of the fovea. This patient had foveal detachment with an outer lamellar hole, a known risk factor for postoperative MH. Notably, this patient presented 4 years later with foveal detachment and an inner lamellar defect in the fellow eye, underwent FSIF ILM peeling, and experienced complete resolution of both the detachment and schisis, with a visual gain of four lines at 3.5 years. The other two MH cases in our study occurred in patients with the MF-only type and the inner LMH type, confirming previous findings that a hole can develop in all types of MF. Additionally, these two patients presented with delayed onset MH after complete peeling at 10 and 24 months, respectively. In such cases, as no recurrent traction was observed, it is possible that the presence of a steep staphyloma and increasing tension on the tangent of the inner retina over time contributed to neurosensory retina opening in an area already weakened by complete peeling. Conversely, in the FSIF group, despite a mean follow-up duration of over 2 years and the presence of 7 out of 22 cases of foveal detachment with retinal thinning at baseline, no cases of postoperative MH were observed. This suggests a potential role for the combined technique in reinforcing foveal structural integrity in the long term and underscores the importance of conducting studies with extended follow-up periods due to the possibility of late onset MH.

In our study, preoperative BCVA was the sole significant predictor of improved BCVA at the final visit. This finding aligns with previously published data indicating that better preoperative vision is the primary factor influencing postoperative BCVA^21^. Our long-term results, which reveal a lower rate of postoperative MH, may encourage earlier intervention in symptomatic patients with relatively good vision.

It is important to acknowledge the limitations of our study. First, it was retrospective and involved a relatively small patient cohort with non-random sampling. Moreover, there was an uneven distribution of patients between groups and a variable follow-up duration within each group. Nevertheless, all patients were followed for a minimum of 12 months, and baseline characteristics were comparable between groups, mitigating selection bias.

In conclusion, our study has demonstrated positive and comparable long-term functional and morphologic outcomes between the fovea-sparing and combined FSIF ILM peeling groups, with the FSIF ILM peeling approach offering additional benefits in preventing MH formation. We advocate for further prospective controlled trials to explore the advantages of combined FSIF ILM peeling in greater depth.

## Methods

This research is a single-center retrospective study that was performed in the Hospital Sacro Cuore–Don Calabria (Negrar, Italy). All methods were approved by the IRCCS Sacro Cuore Hospital Institutional Review Board. The study has been carried out in accordance with the Declaration of Helsinki. Written informed consent was obtained from all participants to participate and publish the identifiable images.

### Patients

In the period of 2012–2020 a total of 36 eyes of 34 consecutive patients with symptomatic myopic foveoschisis underwent vitrectomy. After informing about the procedure and risks of complications patients gave informed consent. Patients were included if they had high myopia (spherical equivalent ≥ − 8.0 D or axial length ≥ 26.0 mm), visual symptoms or decreased vision secondary to MF and a minimum of six months follow-up. Exclusion criteria where full thickness macular hole, choroidal neovascularization, patchy chorioretinal atrophy involving the fovea or previous vitreoretinal surgery.

### Outcome measures

We reviewed the clinical data and demographics of all patients for the following parameters: ophthalmic history, age, sex, lens status, preoperative best corrected visual acuity (BCVA), axial length, spherical equivalent, central foveal thickness (CFT), BCVA at the 1-month follow up and at the last follow-up visit, and postoperative complications. BCVA was measured in Snellen and converted into minimal angle of resolution (LogMAR) for analysis. At baseline axial length was measured with the IOL-master 500 (Carl Zeiss Meditec, Germany).

The eyes were categorized into three sub-groups based on the tomographic pattern of MF: MF-only, MF + inner lamellar macular hole (ILH), MF + foveal detachment (FD).

The CFT was defined as the distance between the inner surface of the neural retina and the inner border of the retinal pigment epithelium and was manually measured using the SD-OCT (Spectralis HR + OCT; Heidelberg Engineering GmbH, Heidelberg, Germany).

After surgery we also assessed the resolution of MF and the integrity of the photoreceptor ellipsoid zone (EZ) at 1-month follow up and last visit. Resolution of MF was defined as the restoration of foveal depression with complete regression of schisis cavities and, if present, foveal detachment. EZ integrity was defined as the visibility of the back-reflection corresponding to the EZ in the foveal scan as a continuous line.

### Surgical procedure

*Standard ILM peeling* A complete 23-gauge vitrectomy was performed. Remnants of vitreous cortex were removed after staining with triamcinolone acetonide. If present, any epiretinal membrane was peeled. The ILM peeling was performed with a mixture of brilliant blue G and trypan blue for 45 s (Membrane Dual; Dorc, The Netherlands). *FSIF peeling*: The procedure was the same as that described above except ILM peeling: the ILM was not completely removed from the retina but peeled off in a circular fashion around the fovea and left attached to the foveal area. The peeled flap was then trimmed with the cutter, using very low aspiration setting to avoid its inadvertent removal. Before fluid-air exchange, low pressure was used to avoid turbulence that could cause a displacement of the flap. Tamponade with 20% sulfur hexafluoride was performed. Most phakic cases underwent combined phacoemulsification and intraocular lens implantation prior to vitrectomy in both procedures.

### Statistical analyses

Descriptive statistics, measures of variability and precision were used to summarize demographic and clinical characteristics, depending on the type of data (continuous or categorical). For continuous data, normality in distribution was tested using Skewness/Kurtosis tests. Two sample t-test for unpaired normal distributed continuous data or its nonparametric correspondent Wilcoxon signed-ranks test were performed to compare means between treatment groups. Two sample paired t-test for normal distributed continuous data or its nonparametric correspondent Wilcoxon matched-pairs signed-ranks test were performed to compare means between two different time point follow-up (baseline vs 1 month and baseline vs last visit). Chi-square or Fisher exact tests were used to compare morphological parameters between the 2 groups (Standard ILM peeling and Fovea Sparing with Inverted Flap ILM peeling). A multivariate linear regression analysis was performed to investigate the linear relation between logMAR BCVA at last visit after surgery (dependent variable) with a set of predictors variables: age, sex, axial length, lens status, type of MF, type of surgery, preoperative CFT, postoperative EZ integrity and follow-up duration. BCVA was measured using the Snellen acuity chart and analyzed it using the logarithm of the minimal angle of resolution (logMAR) scale. Statistical analyses were performed using StataSE software (version 15, College Station, TX). A p-value of < 0.05 was considered for statistical significance.

## Data Availability

The datasets used and/or analysed during the current study are available from the corresponding author on reasonable request.
